# Spontaneous Resolution of Carotid Stenosis with Free Floating Thrombus: A Brief Overview of Possible Mechanisms and Management

**DOI:** 10.7759/cureus.7602

**Published:** 2020-04-09

**Authors:** Andre Monteiro, Yasmin Cunha, Gustavo M Cortez, Eric Sauvageau, Ricardo Hanel

**Affiliations:** 1 Neurological Surgery/Stroke Neurology, Baptist Neurological Institute/Lyerly Neurosurgery, Jacksonville, USA; 2 Neurological Surgery/Stroke Neurology, Baptist Neurological Institute, Jacksonville, USA; 3 Neurosurgery and Cerebrovascular Diseases, Baptist Neurological Institute/Lyerly Neurosurgery, Jacksonville, USA; 4 Neurological Surgery, Baptist Neurological Institute/Lyerly Neurosurgery, Jacksonville, USA; 5 Neurological Surgery, Baptist Neurological Institute/lyerly Neurosurgery, Jacksonville, USA

**Keywords:** carotid stenosis, free floating thrombus, atherosclerosis, carotid artery, plaque rupture, plaque erosion

## Abstract

Free floating thrombus is uncommon in the carotid arteries, and it is defined as an elongated thrombus attached to the arterial wall with circumferential blood flow at its distal most aspect. The majority of patients present with acute neurological deficits and usually have an underlying unstable atherosclerotic plaque. We present two cases of carotid stenosis associated with free floating thrombus. Both patients shared similar clinical presentation and were managed according to the standard workflow for suspected ischemic strokes from our institution. Conservative management was decided based on a multidisciplinary decision that intervention of any kind would be associated with high risk of periprocedural complications. Complete resolution of the atherosclerotic plaque and free floating thrombus in a short period occurred. We further discuss the mechanism behind such a dramatic radiographic and clinical improvement with medical therapy, hypothesizing that plaque erosion is a plausible explanation, based on pathology studies.

## Introduction

Unstable and vulnerable plaque with free floating thrombus (FFT) in the carotid circulation is an uncommon finding. Since the first possible report in 1905, a definition for this event remained divergent for many years, until a review by Bhatti et al. of less than 150 reported cases reported in the literature suggested a concise concept: an elongated thrombus attached to the arterial wall with circumferential blood flow at its distal most aspect [1,2]. This phenomenon was associated with atherosclerotic plaques in the internal carotid artery (ICA) in most cases and found to be more frequent in men [1]. The majority of patients with FFT are symptomatic and revealed to be a great challenge from the decision-making point of view, with some authors stating that it should be immediately repaired through surgery, while others formulate medical therapy alone as a safer choice [3,4]. However, it remains unclear whether surgical management is superior to medical therapy alone [1]. We postulate that the origin of these sessile thrombus to be related to plaque erosion and report two cases managed medically.

## Case presentation

Case 1

Clinical Presentation and Assessment

A 57-year-old male was admitted to the emergency department 12 hours after sudden onset of left lower extremity weakness. Past medical history was relevant for ischemic stroke six months prior to admission with residual left upper extremity weakness, deep vein thrombosis and inferior vena cava filter placement. Baseline modified Rankin score (mRs) was 1. At examination, there was complete hemianopia, facial droop, left lower extremity decreased sensation and weakness. National Institutes of Health Stroke Scale (NIHSS) was 6. He was supposed to be on anticoagulation therapy but had not been taking warfarin for the past two weeks.

**Figure 1:**
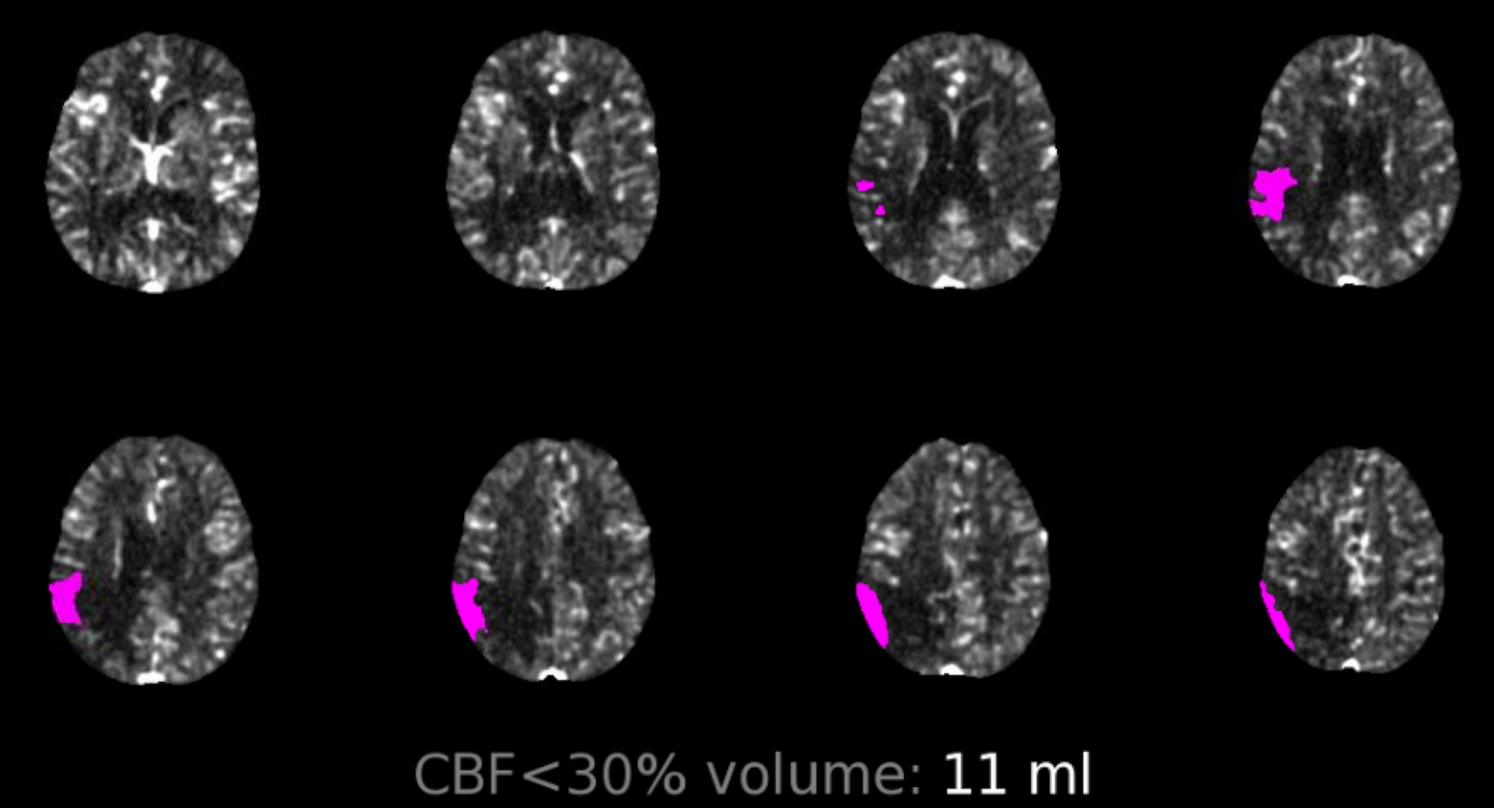
Computed tomography perfusion imaging of case 1 Computed tomography perfusion showing small acute cerebral infarction in the middle cerebral artery territory, with overall infarct volume of 11 mL, based on cerebral blood flow (CBF) less than 30% (RAPID software; iSchemaView Inc., Menlo Park, CA)

Initial assessment confirmed that he was out of therapeutic window (international normalized ratio [INR] was 1.1). Computerized tomography perfusion (CTP) was positive for a small acute cerebral infarction of middle cerebral artery cerebral artery territory (Figure 1). Cardiac source of embolism was unlikely after negative ECG for atrial fibrillation and no obvious thrombus displayed on echocardiogram.

**Figure 2:**
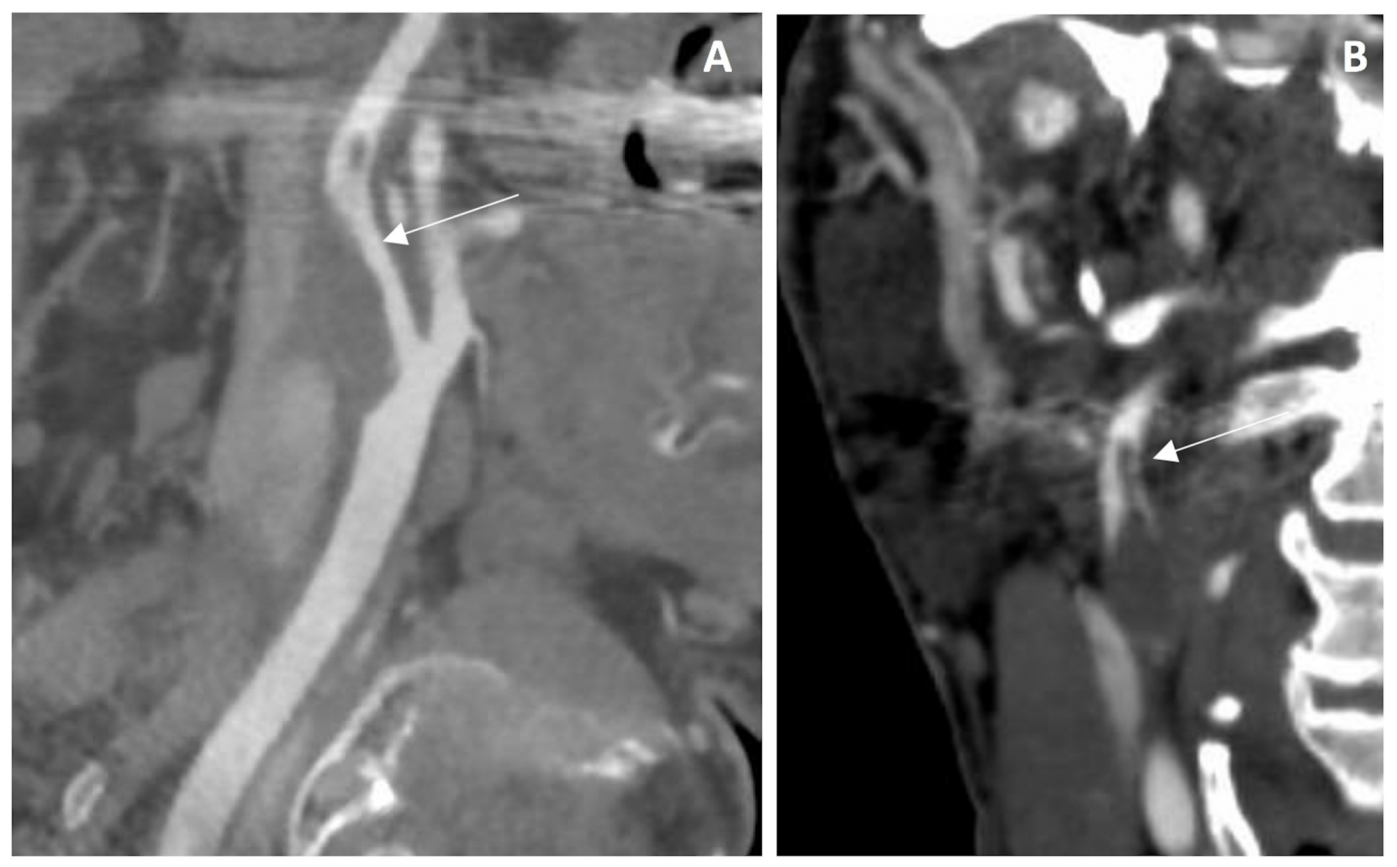
Computerized tomography angiography initial assessment of case 1 Computerized tomography angiography sagittal view of the right internal carotid artery showing hypodense plaque causing significant lumen stenosis (A), and coronal view of the right internal carotid artery demonstrating elongated free floating thrombus (B).

Computerized tomography angiography (CTA) of the head and neck revealed an intraluminal thrombus arising from a hypodense plaque in the proximal segment of the right ICA, resulting in 54% of lumen stenosis, according to the NASCET criteria (Figure 2). Low attenuation of the plaque imaging was suggestive of soft lipid-rich material, which is known to be an unstable feature. Final diagnosis was acute ischemic stroke and symptomatic moderate carotid stenosis of atherosclerotic etiology. 

Management and Outcome

The patient was out of time window to benefit from intravenous tissue plasminogen activator administration. Endovascular or surgical intervention at this point was not considered due to the nonocclusive nature of the thrombus, as well as a high risk of further distal embolization. Decision was then made to manage the case conservatively. He was started on heparin drip, warfarin and aspirin, and was admitted to the neurocritical care unit for close monitoring. Within three days from presentation, the patient’s symptoms gradually improved. Repeated CTA five days after admission revealed residual right ICA stenosis and partial resolution of the intraluminal thrombus.

**Figure 3:**
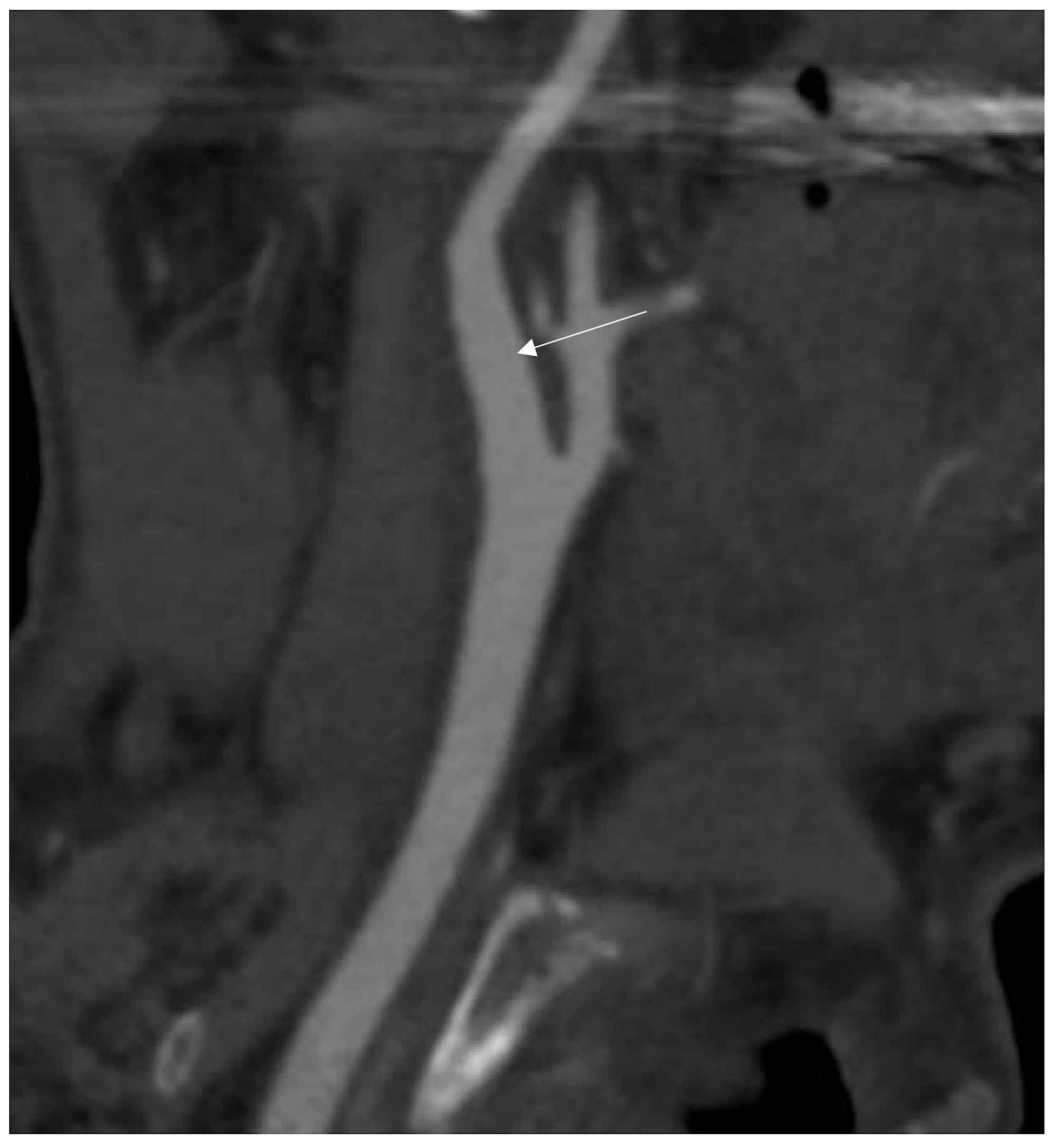
Computed tomography angiography follow-up of case 1 Computed tomography angiography sagittal view on 24-day follow-up showing complete resolution of the carotid plaque

The patient was discharged home 10 days after being admitted. He had mild left lower extremity weakness and residual neurological deficits that were already present prior to this ischemic event. Discharge NIHSS and mRs were both 2. On the last day of his hospital stay, his INR was 2.4 and he was sent home on warfarin 15 mg and aspirin 325 mg daily. CTA performed 24 days after initial presentation revealed no significant degree of stenosis and complete resolution of intraluminal thrombus in the right ICA (Figure 3). 

Case 2

Clinical Presentation and Assessment

A 30-year-old male presented to the emergency department with sudden onset right upper extremity paresthesia and altered speech. Prior medical history was relevant for previous percutaneous coronary intervention under optimized medical therapy, which included dual antiplatelet regimen, statin and beta-blocker. Baseline mRs was 0. At admission, blood pressure was 116/70 mmHg. During neurological examination, there was decreased sensation in the right upper extremity and speech was intact (NIHSS 1).

**Figure 4:**
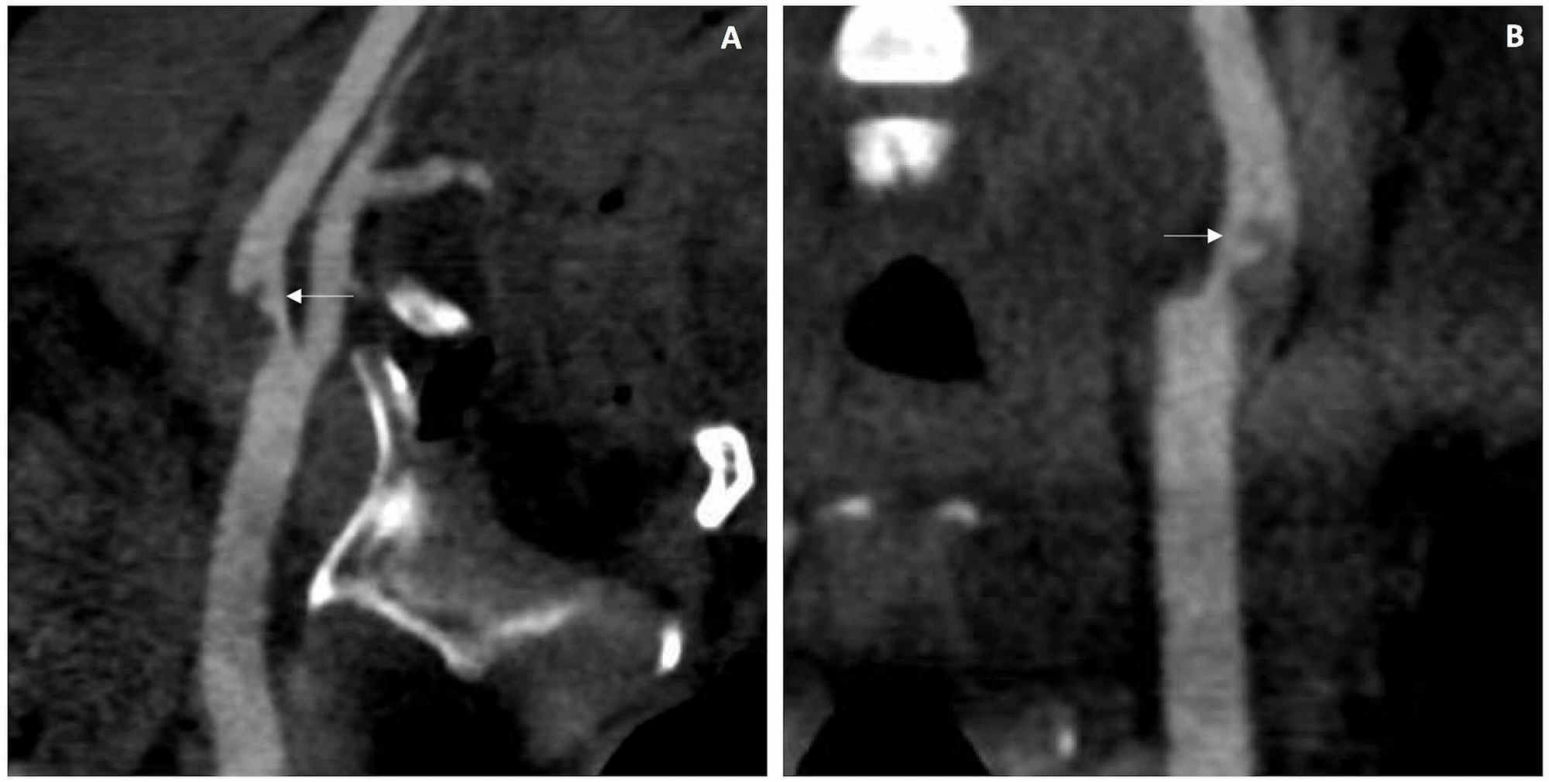
Computerized tomography angiography initial assessment of case 2 Computerized tomography angiography sagittal view of the left internal carotid artery demonstrating hypodense plaque with short intraluminal extension causing significant lumen stenosis (A), and coronal view of the right internal carotid artery evidentiating curved shape of the free floating thrombus (B)

Initial assessment to investigate antiplatelet response revealed P2Y12 levels of 200 PRU. CTP was negative for cerebral infarction. CTA of the head and neck demonstrated a hypodense plaque in the left ICA causing 77% of diameter reduction, according to the NASCET criteria, and a curved short intraluminal thrombus (Figure 4). The hypodensity of the plaque was suggestive of soft lipid-rich material.

**Figure 5:**
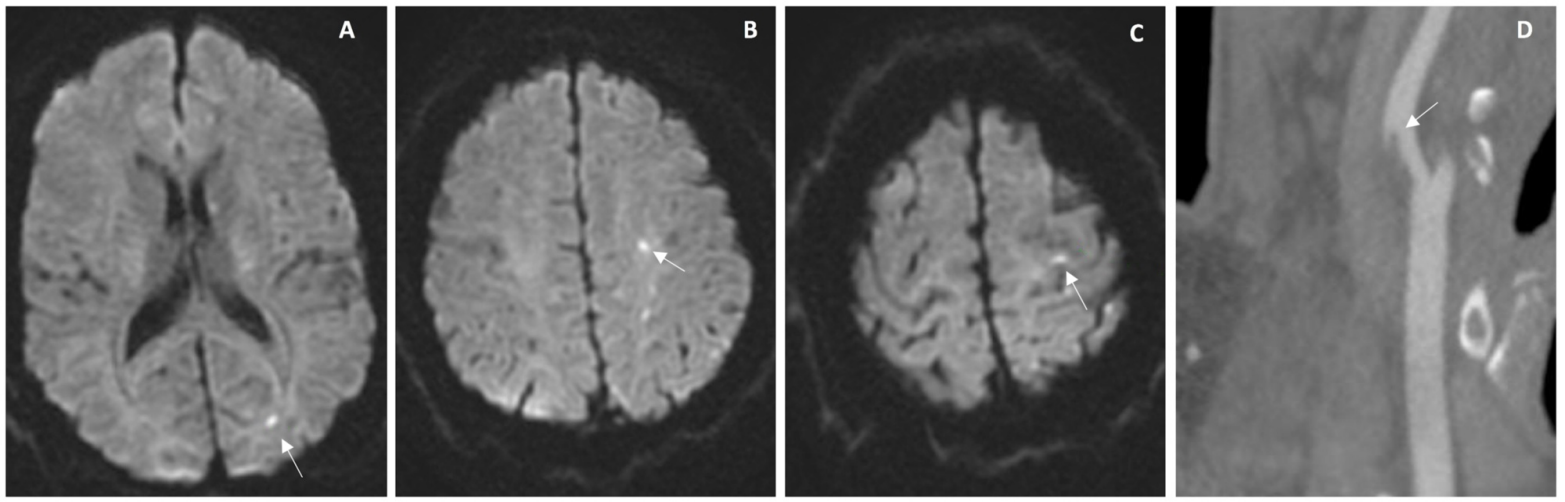
Imaging studies performed during hospital stay of case 2 Studies performed 48 hours after admission were MRI showing acute punctual infarctions (A-C) and computed tomography angiography demonstrating no change in the baseline degree of stenosis or intraluminal thrombus resolution (D)

After improvement in symptoms within the next day, a MRI and another CTA were performed 48 hours after admission, demonstrating small acute cerebral infarctions (Figures 5A-5C) and no change in the baseline degree of stenosis or intraluminal thrombus resolution (Figure 5D). The final diagnosis was acute ischemic stroke and symptomatic severe carotid stenosis. 

Management and Outcome

The patient was admitted to the neurocritical care unit and started on heparin drip, maintaining his previous antiplatelet medications. Our multidisciplinary team decided to maintain close neurological monitoring until radiographic stabilization.

**Figure 6:**
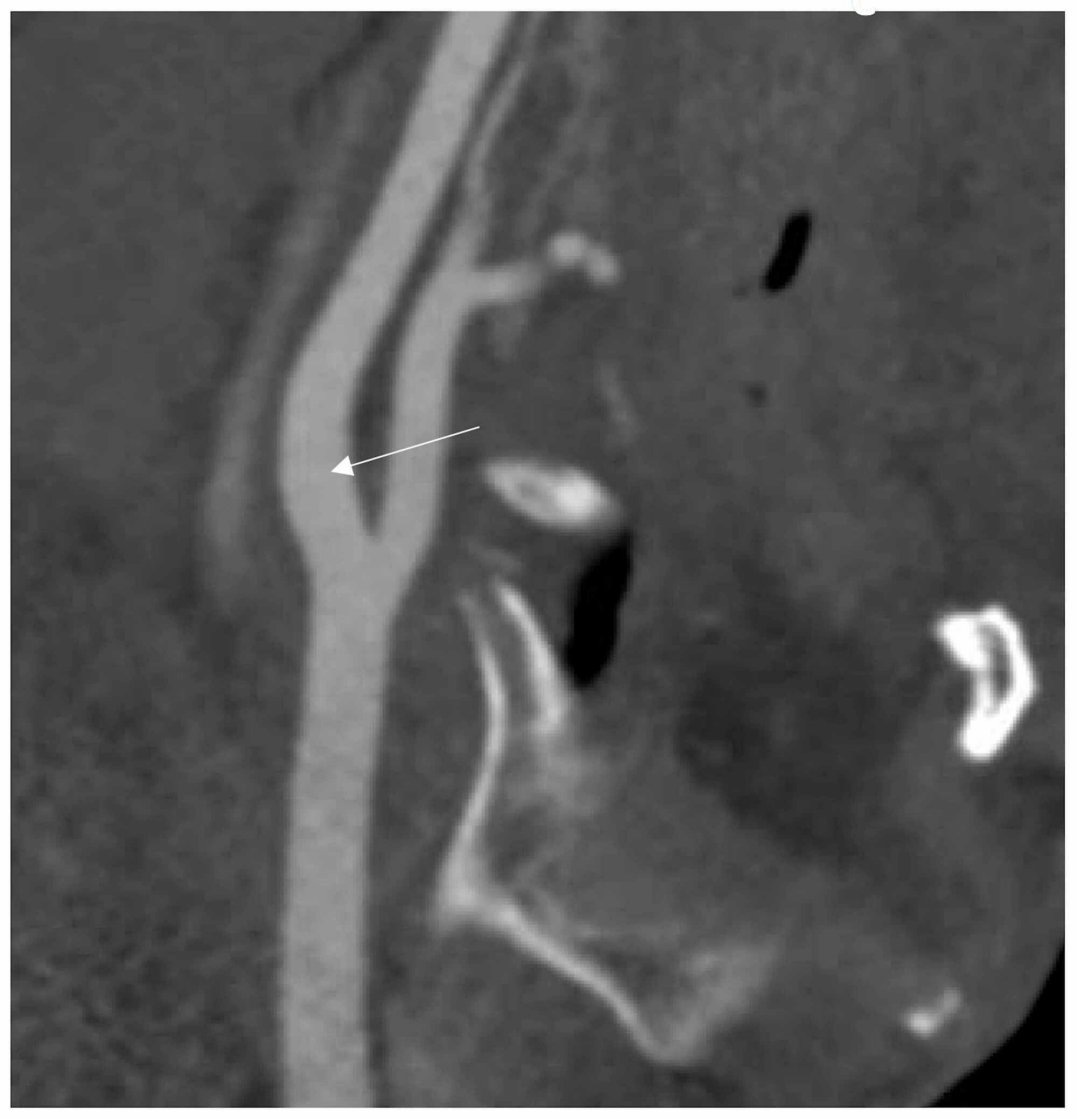
Computed tomography angiography follow-up of case 2 Computed tomography angiography sagittal view on 14-day follow-up showing complete resolution of the carotid plaque

Unfortunately, the patient insisted in obtaining discharge. The team discussed all the risks of his condition and he agreed. He was discharged home on new antiplatelet regimen, aspirin 325 mg and clopidogrel 75 mg daily, and scheduled to be seen again within two weeks. CTA performed 14 days after initial presentation showed no significant degree of stenosis and total resolution of intraluminal thrombus in the left ICA (Figure 6). The patient was asymptomatic and mRs was 0.

## Discussion

Presence of soft plaque with rupture or erosion with associated FFT is not a common finding in the carotid arteries. The largest review by Bhatti et al. reported that this entity was found more frequently in men (2:1), and the patients presented with acute neurological deficits in 92% of cases. The most common location was the ICA and it was not associated with atherosclerotic plaques in only a third of the articles [1]. However, it remains unclear what is the most appropriate management for FFT. Conservative treatments reported for this phenomenon included anticoagulation and antiplatelet therapy, both combined or alone [5]. Surgical approaches found in literature were carotid angioplasty and stenting (CAS) and early or delayed carotid endarterectomy (CEA) [6,7]. Although FFT cases in literature required an individualized decision making, it seems that they were handled mostly according to the carotid atherosclerotic disease present, at least in cases where it was found an association of both, which were the majority. 

Indication for carotid revascularization for both symptomatic and asymptomatic carotid artery stenosis has been standardized for many years. Nevertheless, some plaque features, anatomical location and timing of surgery have been well correlated with limitations and appropriate use of each modality. CEA is safe during the first two weeks after acute neurological events. It can be considered as an emergency intervention to prevent further embolic events in patients with pre-occlusive disease and small infarction core with large penumbra area, which was not the case in any of our patients [8]. Carotid lesions located above the level of C2 vertebra and below the clavicle are difficult to access surgically and consequently can increase the morbidity of CEA [8]. Soft lipid-rich plaques with intraluminal thrombus are associated with high risk of periprocedural stroke when submitted to CAS [9].

Regarding the pathophysiology, we hypothesize that plaque erosion, rather than rupture, is the more likely mechanism behind FFT in our cases. These two concepts were extensively discussed in several pathology studies of carotid and coronary atherosclerotic plaques [10-12]. Plaque rupture is defined as complete disruption of the fibrous cap and direct contact of the luminal thrombus with the lipid core. Plaque erosion is defined as de-endothelization of the plaque, in which the thrombus has contact with the cap’s subendothelial tissue, but not with lipid core [10-12]. In morphological studies of carotid plaque in patients who underwent CEA, plaque erosion was not frequently found in those with previous stroke, but it had higher prevalence in individuals who suffered transient ischemic attack [10,13]. Although these studies suggest that erosion is an uncommon cause of stroke, we believe that a fast and complete resolution of the plaque, as observed in the current reports, would not happen in the setting of fibrous cap rupture and exposure of the necrotic lipid core to the lumen, which is expected to cause more significant thrombosis and severe clinical presentation.

Most cases of FFT in literature were diagnosed with digital subtraction angiogram (DSA), which is attributable to a historical bias of times when CT and MR-based images were not widely available. However, DSA remains the gold standard method to evaluate carotid stenosis and it is considerably safe when performed in adequate cases and by a skillful operator. Our patients were diagnosed and managed with noninvasive imaging only, and it yielded an excellent visualization of the present carotid disease and the FFT without any risks added. Although DSA and CTA are helpful to demonstrate the degree of luminal stenosis, they are limited when it comes to assessment of other features associated with recurrent symptoms. MRI of carotid atherosclerotic disease is a method that has promising results to reveal inflammation, another predictor indicative of symptomatic plaques, especially in those without hemodynamic repercussion (low to moderate grade stenosis) [14]. Currently, the main limitation of this imaging technique as a screening tool is economic, due to the high prevalence of low and moderate grade stenosis, but it is an excellent choice for symptomatic individuals [14]. Studies have been investigating biomarkers which could be used as screening of asymptomatic patients to assess the risk of developing ischemic symptoms [15]. This could narrow the target population for MR plaque imaging and increase its role in carotid atherosclerotic disease [15,16].

## Conclusions

FFT in atherosclerotic plaques is an uncommon event in the carotid arteries. The clinical presentation with acute neurological deficits requires effective management and decision to either perform an interventional procedure or to treat with medical therapy alone, but there is no consensus regarding best treatment in these cases. We witnessed complete radiological and clinical recovery of two patients and hypothesized that the mechanism of FFT could be due to plaque erosion instead of rupture, according to pathology studies in literature. Noninvasive imaging, such as CTA, is a useful tool in the initial assessment and follow-up of unstable carotid plaques, and MR plaque imaging is a promising tool that should become more available in the future.

## References

[1] Bhatti AF, Leon LR Jr, Labropoulos N (2007). Free-floating thrombus of the carotid artery: literature review and case reports. J Vasc Surg.

[2] Ehrenfeld WK, Hoyt WF, Wylie EJ (1966). Embolization and transient blindness from carotid atheroma. Surgical considerations. Arch Surg.

[3] Ferrero E, Ferri M, Viazzo A (2011). Free-floating thrombus in the internal carotid artery: diagnosis and treatment of 16 cases in a single center. Ann Vasc Surg.

[4] Lane TR, Shalhoub J, Perera R (2010). Diagnosis and surgical management of free-floating thrombus within the carotid artery. Vasc Endovascular Surg.

[5] Mathew S, Huntley L, Sowinski A (2002). Mobile carotid artery thrombus: is it a surgical emergency?. Eur J Vasc Endovasc Surg.

[6] Buchan A, Gates P, Pelz D, Barnett HJ (1988). Intraluminal thrombus in the cerebral circulation. Implications for surgical management. Stroke.

[7] Chakhtoura EY, Goldstein JE, Hobson RW (2003). Management of mobile floating carotid plaque using carotid artery stenting. J Endovasc Ther.

[8] Ricotta JJ, Aburahma A, Ascher E, Eskandari M, Faries P, Lal BK (2011). Updated Society for Vascular Surgery guidelines for management of extracranial carotid disease. J Vasc Surg.

[9] Setacci C, Chisci E, Setacci F, Iacoponi F, de Donato G, Rossi A (2010). Siena carotid artery stenting score: a risk modelling study for individual patients. Stroke.

[10] Spagnoli LG, Mauriello A, Sangiorgi G (2004). Extracranial thrombotically active carotid plaque as a risk factor for ischemic stroke. JAMA.

[11] Farb A, Burke AP, Tang AL (1996). Coronary plaque erosion without rupture into a lipid core. A frequent cause of coronary thrombosis in sudden coronary death. Circulation.

[12] Carr S, Farb A, Pearce WH, Virmani R, Yao JS (1996). Atherosclerotic plaque rupture in symptomatic carotid artery stenosis. J Vasc Surg.

[13] Redgrave JN, Lovett JK, Gallagher PJ, Rothwell PM (2006). Histological assessment of 526 symptomatic carotid plaques in relation to the nature and timing of ischemic symptoms: the Oxford plaque study. Circulation.

[14] Wasserman BA, Wityk RJ, Trout HH III, Virmani R (2005). Low-grade carotid stenosis: looking beyond the lumen with MRI. Stroke.

[15] Peeters W, Hellings WE, de Kleijn DP (2009). Carotid atherosclerotic plaques stabilize after stroke: insights into the natural process of atherosclerotic plaque stabilization. Arterioscler Thromb Vasc Biol.

[16] Virmani R, Finn AV, Kolodgie FD (2009). Carotid plaque stabilization and progression after stroke or TIA. Arterioscler Thromb Vasc Biol.

